# Predisposing and Motivational Factors Related to Social Network Sites Use: Systematic Review

**DOI:** 10.2196/12248

**Published:** 2019-06-09

**Authors:** Elisa Mancinelli, Giulia Bassi, Silvia Salcuni

**Affiliations:** 1 Department of General Psychology University of Padua Padua Italy; 2 Human Inspired Technology Department of Developmental and Socialization Psychology University of Padua Padua Italy; 3 Department of Developmental and Socialization Psychology University of Padua Padua Italy

**Keywords:** social networks, individual differences, motivation, adolescents, adults

## Abstract

**Background:**

Social network sites (SNSs) have been defined as Web services that involve creating a private or semiprivate profile. Through these services, adolescents and adults can maintain and create new relationships. Adolescents, in particular, can be considered the main users of these sites as they spend a lot of time on SNSs. In using SNSs, individuals can exert greater control over the conversation and on the information shared, which is associated with a desire for self-presentation. Moreover, the need for self-presentation is related to personality traits such as those of the Big Five, namely extraversion, neuroticism, openness to experience, agreeableness, and conscientiousness, as well as emotional stability, introversion, narcissism, and motivational aspects. The latter are usually linked to an underlying social purpose that might predispose an individual to using SNSs, with the intent of satisfying particular needs, such as belongingness and interpersonal competency.

**Objective:**

Using the Preferred Reporting Items for Systematic Reviews and Meta-Analyses (PRISMA) method, this study aimed to present a systematic review of the scientific literature regarding the predisposing factors related to the Big Five personality traits and motivational aspects associated with the use of SNSs, for both adolescents (12-19 years) and adults (>20 years).

**Methods:**

A search ranging from 2007 to 2017 was conducted through the academic database of Google Scholar and PsycINFO, in which the following terms and their derivatives were considered: *predisposing factors, personality traits, Big Five model, self-esteem, self-presentation, interpersonal competency, social network site, Facebook, motivation, five-factor model, use, abuse,* and *addiction.* Based on a defined list of inclusion and exclusion criteria, a total of 9 papers were finally included in the review.

**Results:**

Our findings identified 3 main personality traits to be of greater value: extraversion, neuroticism, and openness to experience. Extraversion was a good predictor of motivation and SNS use, whereas the latter trait showed relevance for age differences. All 3 features further played a role in gender differences. Apart from extraversion, the self-presentational motive was also related to narcissism, whereas the need to belong presented an association with agreeableness and neuroticism. Further underlining the social value behind SNS use, people perceived interpersonal competency as being related to Facebook use intensity.

**Conclusions:**

Extraversion was recognized as the main forerunner for SNS use and motivation for use. Neuroticism seems to be related to an attempt at compensating for difficulties in real-life social contexts. Openness to experiences has a strong valence for both adults and older adults since SNSs are still perceived as a novelty. Moreover, gender differences in SNS usage were observed to be the product of differences in motivation. Implications and limitations of the study were discussed.

## Introduction

### Background

Social network sites (SNSs) have been defined as Web services that allow creating a private or semiprivate profile, a list of contacts, and that gives the possibility to scroll down the list of one’s Web friends and to communicate with them [1]. Through these services, adolescents as well as adults can maintain and create new relationships. In particular, adolescents can be considered as the main users of these sites as they spend a lot of time on SNSs. Indeed, it is possible to affirm that such sites (eg, Facebook) have a great impact and role on youngsters’ lives and, as affirmed in the study by Brown [2], this population can thus be defined as *the new media generation*. For example, these Web services allow individuals to think longer about their own answers while also pondering more on how to express themselves, therefore, exerting greater control on the conversation and on the information shared, which is all associated with a desire for self-presentation [2]. Moreover, the need for self-presentation is related to personality traits such as those of the Big Five, namely extraversion, neuroticism, openness to experience, agreeableness, and conscientiousness, together with emotional stability, introversion, and narcissism. Personality traits are important also for the motivational aspects, usually related to an underlying social purpose that might predispose an individual to using SNSs with the intent of satisfying their particular needs (eg, self-presentational motives, belongingness needs, and interpersonal competency) [2,3]. Furthermore, in relation to individual predispositions in the frequency of SNS use, an additional role is played by gender and age. Indeed, a relation between gender differences, motivation, personality traits, self-esteem, and specific SNS usage patterns was observed, thus not only considering the broader frequency of SNS use.

### Self-Presentation and the Big Five Personality Traits

Self-presentation is a process through which individuals present themselves to the social world, and it is usually motivated by a desire to please others [3]. Self-presentation can be used as a means to manage the impressions of others relatively to oneself while also behaving in different ways, thus creating the desired impressions [3]. For example, an individual can interact with many people *vis-à-vis* throughout the day and create different impressions on each person. In particular, considering personality, neuroticism refers to a state of negative emotionality characterized by feelings of anxiety, depression, vulnerability, and angry hostility, whose counterpart is emotional stability. As a second factor, openness to experience describes a person that is fantasy prone, who values curiosity, and who shows behavioral flexibility. A subsequent dimension is that of conscientiousness, representing an individual that is achievement striving, self-disciplined, and conscientious. Agreeableness is instead associated with altruism, compliance, and tender-mindedness. Finally, the trait extraversion defines an individual that is assertive, highly sociable, and who shows positive emotions and impulsivity, whose opposite dimension is introversion [4]. Nowadays, impressions on people can also be transmitted through the use of SNSs in which individuals can control the conversation and the information shared, thus appearing to be related to a desire for self-presentation. In particular, it could be interesting how this desire for self-presentation might be associated with personality traits, as the above-mentioned dimensions, thus those of the Big Five personality traits, when mediated by the use of SNSs or in the context of SNSs interactions. However, greater empirical support is needed to better understand the link between self-presentation, the Big Five personality traits, and SNS use.

### Motivational Aspects: The Need for Self-Presentation, Need to Belong, and Interpersonal Competency

Motivation can be defined as *a reason or reasons for acting or behaving in a particular way*, thus, as something energizing one’s behavior toward a specific action, independently from one’s character, highlighting its value when trying to comprehend a certain demeanor [5]. Through motivation, it is possible to observe that individuals, all with specific characteristics, present certain needs, which are then satisfied by employing them as motivators [5]. Furthermore, this factor is of even greater value as it might be more easily assessed than the broad category of personality traits as described by the Big Five with consequences on treatment and clinical interventions. Specifically, motivation will be investigated in relation to the Big Five personality traits and narcissism, particularly considering interpersonal competency motivation, which focuses on individuals’ ability to interact on the Web and in real-life with others, the need of belongingness, and the self-presentational motive. In particular, the *need for self-presentation* specifies a behavior aimed at continuously monitoring the impression we would like to give on to people, whereas the *need to belong* refers to an individual necessity to affiliate with others and to seek social contacts [3]. However, greater empirical support is needed to better understand the 3 motivational components considered.

### The Role of Gender and Age in Social Network Sites Use

The relation between motivation, personality traits associated with the interpersonal competency, the need to belong, the need for self-presentation, and to age-gender differences in real life, is widely provided [6-8]. For example, it has been shown that generally females, in respect to males, confer greater priority in creating a positive self-impression, whereas males are less worried about the image they present in face-to-face interactions [6]. Indeed, during such real-life encounters, women have been recognized to show, on average, a more agreeable and nurturing demeanor than men do [7]. A similar dynamic can also be observed in self-presentational behavioral differences among people of different age groups when communicating with others *vis-à-vis*. In line with the above considered gender differences example, adolescents and young adults are reported to be less agreeable and conscientious than older adults, which is probably because of the specific developmental stage the formers are going through [8]. As a matter of fact, both traits of conscientiousness and agreeableness tend to increase throughout early and middle adulthood. Still, considering SNS use in this context, its role as defined by past literature is not always clear. Furthermore, in such instance, as for age and gender differences are concerned, self-esteem could be otherwise speculated of being of greater value in SNS use. Indeed, self-esteem showed a relationship with life satisfaction, academic success, social relationships, and mental and physical health; thus, self-esteem could also be an important motivator for SNS use [9]. Therefore, the variables considered in the analysis of data are the cause of the great variation in the results obtained, leading to the inconsistent results observed.

The aim of this study was to conduct a systematic review of the scientific literature concerning the predisposing factors related to the Big Five personality traits and motivational aspects associated with SNS use in general, and therefore, trying to define a comprehensive outline of the past literature on the matter. The intent is to understand on SNSs *who* and subsequently *how* behaves in a certain manner, instead of another, particularly as these sites have become increasingly salient in people’s life experiences already from a young age.

## Methods

### Search Process

Following the PRISMA Group workflow [10], a systematic literature review from 2007 to 2017 has been conducted through the academic database of Google Scholar and PsycINFO, in which the following terms and their derivatives were considered: *predisposing factors, personality traits, Big Five model, self-esteem, self-presentation, interpersonal competency, social network site, Facebook, motivation, five-factor model, use, abuse,* and *addiction.*

### Inclusion Criteria

The articles selected for the subsequent analysis were in line with the following inclusion criteria: (1) specificity to SNS use (with a focus on Facebook); (2) predisposing features related to the Big Five personality traits as reported by the five-factor model (FFM) or Big Five and narcissism; (3) motivational aspects connected to the need for self-presentation, need to belong, and interpersonal competency; (4) the role of self-esteem; and (5) the role of gender and age (adolescents, 12-19 years; adults, >20 years) in self-esteem for SNS use.

### Exclusion Criteria

Studies that met any of the following criteria were excluded: (1) internet use in general and blogging; (2) the consequences of SNSs and internet use; (3) the relation between attachment style and SNS use; and 4) the association between psychological disorders and the type of SNS use. Independently, 2 rates judged the papers on inclusion-exclusion criteria (PRISMA workflow; [10]).

## Results

### Study Characteristics

**Figure 1:**
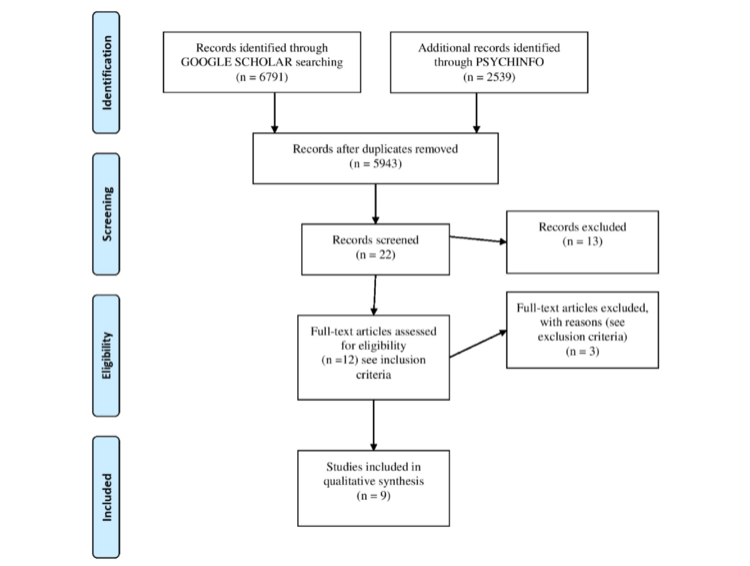
Preferred Reporting Items for Systematic Reviews and Meta-Analyses chart summarizing the selection process.

As shown in Figure 1, final results highlighted 9 studies (2 reviews and 7 types of research) regarding adolescents (12-19 years) and adults (>20 years). Gender and age differences have been considered as fundamental in SNS use particularly, considering the predisposing features related to the Big Five personality traits and analyzing the need for self-presentation, the need to belong, and interpersonal competency. Moreover, Multimedia Appendix 1 summarizes the main characteristics of the included studies. A total of 2230 participants were included in five studies [11-15], of which were conducted on young adults. As for the review [1], it took into account 42 articles concerning young adults. Two papers [16,17] considered both young adults and adolescents, one study [16] was carried out on 463 subjects whereas the other [17] reviewed 42 articles. The remaining one [18] was conducted on 275 adolescents. Four of the studies [12,13,16,18] focused on the Big-Five personality traits; 3 [11,14,17] on the relation between Big-Five and motivational factors for Social Networks use; and 1 study [1] targeted the relation between Big-Five, motivational factors and self-esteem. The remaining one [15] only considered self-esteem and motivation. All studies used and referred to self-reports to measure the outcomes.

### Social Network Sites Use and the Big Five Personality Traits

As already stated, of greater relevance is the personality trait extraversion, as it has been widely recognized as the dimension better accounting for SNS use; still, results are contradictory. Indeed, no association was reported between extraversion and the amount of *time spent on Facebook* [11], whereas later research [16] observed that such trait was associated with the intensity of Facebook use over and above all other Big Five personality factors and gender. Thus, supporting the previous study in which extraversion was found to be the strongest predictor of SNS use in general [12].

One research [11] highlighted that extraversion was related to greater group membership but not significantly related to the *number of friends on Facebook.* This was explained by suggesting that extraverts use SNSs as an addition to their social life and not as an alternative to it. Different results were although reported, where extraversion was observed, together with gender, to significantly predict the number of friends on SNSs, where females presented more friends than the male counterpart [13]. Moreover, results assessed that once age, gender, and school grade were controlled for, the analyzed personality trait accounted for the number of friends over and above narcissism [18]. However, only extraversion accounted for the influence on the *frequency of SNS use,* whereas neuroticism seems not to be particularly predictive [16].

Contradictorily, in previous findings [12] considering emotional stability (low neuroticism), it was reported that those on this side of the continuum used SNSs less frequently, implying that the more anxious and worrisome individuals (thus, high on neuroticism) are those more drawn to the use of SNSs. A study [11] considering the amount of *personal information shared* on SNSs observed neuroticism to be unrelated to the posting of such information as explained by findings in which neurotic individuals were reported to be more controlling of the information shared [14]. Results further showed that those high on neuroticism preferred posting on their personal wall as compared with sharing photos, probably because of the possibility of better manipulating the content shared. Contrarily, another study [11] hypothesized and confirmed for neuroticism to be positively related to different self-aspects (*actual-self, hidden-self,* and *ideal-self*), together with *general* and *emotional self-disclosure*, with the latter being mediated by all the self-aspect and thus supporting past literature stating the value of self-presentation in the sharing of personal information and emotions [13].

As for what conscientiousness is concerned, these individuals being highly duty focused, most research considering its relation with SNSs proposed a negative association, assuming that SNSs would be perceived as a distraction. However, differently from past literature, the negative relation was not supported, and instead, no relation was reported [10,12]. Therefore, extraversion appears to be the strongest predictor of SNS use and motivation for use in general, over and above all other Big Five personality traits, followed by neuroticism, and then by openness to experience, for which age differences are glaring. Regarding the other personality traits, further research could be prompt so to better understand the role of agreeableness and conscientiousness in relation to SNS use. Particularly, these last 2 seem to have been only partially considered or not considered at all, which might be because of the marginal association reported with SNSs by past literature.

### The Need to Belong and Social Network Sites Use

A study related to the dual-factor model, with a sample of American young adults (mean age=19.51 years), showed that for the *need to belong* its best predictors are high agreeableness and high neuroticism, for antipodal reasons [1]. In particular, the latter expresses a difficulty in social contacts and relations, leading neurotic individuals to try and fulfil their cohesion needs in Web platforms, whereas the former defines a person that is friendly and compliant and that, as such, shows great belonging motives, explaining why this trait is a strong predictor of belongingness on SNSs, being particularly associated with belonging to groups. Supporting this, it has been observed that individuals high in group identification and positive collective self-esteem felt as the most important motivator for SNS use the maintenance of contacts with their close group of friends [15]. Such a difference between findings can be explained by the way in which the need to belong is defined and assessed. In line with belongingness needs, the value of neuroticism in this instance lies in neurotic people attempt of compensating for their inability to optimally self-present themselves in a face-to-face encounter, thus, allowing them to show also their hidden and ideal self-aspects. Low conscientiousness is instead straightforwardly explained by the fact that those high on this dimension exert greater cautiousness when presenting themselves, especially on the Web, preferring an authentic self-presentation.

### Self-Presentational Motives and Social Network Sites Use

Considering the dutifulness encompassing the trait conscientiousness, the effort of better presenting themselves on the Web could be expected to be perceived as un unnecessary waste of time, reason for which individuals lacking on such trait tend to self-present themselves more. Indeed, it was observed that high neuroticism was predictive of *self-presentational needs*, together with low conscientiousness [14]. One study regarding self-presentational needs showed narcissism to be of greater relevance but only for self-generated content (ie, rating of one’s profile pictures and frequency per week of status updates) compared with system-generated content (ie, the number of Facebook friends and photos) [18]. Past research [17,18] reported that narcissists have a larger Web social network, which in the study was instead accounted, together with photo count, by extraversion; thus, it allows to assume that people high on narcissism may have not yet started using such features as self-presentational means. This contradiction between findings can be explained by the age of the sample [18]. Adolescence is a period in which social life is yet to be fully developed, as opposed to university students for which social life is usually at its highest [18]. Such observation considers age as a limitation, although fostering the idea of comparing samples of different ages, with the aim of observing how narcissism translates on SNSs in different periods of life.

### Interpersonal Competency and Social Network Sites Use

A further motivational element that needs to be reported is that of *interpersonal competency.* From past literature, a study by Jenkins et al [16] identified 2 domains relevant in Web social behavior used to define *interpersonal competency* (together with disclosure, negative assertion, and conflict management, which were not considered as variables): *initiating relationships* and *emotional support*. This same study [16], concerned with the relevance of personality and self-esteem in one’s felt interpersonal competency and its subsequent impact on the frequency of Facebook use, underlines the importance of the previously mentioned research [18], as “the new media-generation” [2,16] has transported on the Web the developmental stages related to social behavior and cohesion once held in real-life [16,19]. In this regard, results showed that the intensity of Facebook use was related to perceived interpersonal competency. Specifically, individuals used this element as a motivator to compensate for their felt lack in relation to the initiation of relationships, although not accounting for emotional support. Interestingly, the 2 domains of interpersonal competency were not related to neuroticism, which could be intuitively associated with being shy and interpersonally incapable, together with the fact that neurotic individuals, as also previously discussed in relation to belongingness, were observed to use Web platforms to compensate for their lack of real-life contacts [14].

### Age Differences in Social Network Sites Use

In a 2010 experiment, extraversion, emotional stability, and openness to experiences were adopted as personality variables, further accounting for life satisfaction in age differences [13]. The young cohort (from 18-29 years) frequency of SNS use was only predicted by extraversion, whereas the other 2 traits were found to be not significantly related with it, with life satisfaction never playing a significant role especially after accounting for personality.

As for the older ones (older than 30 years), all 3 personality dimensions showed a predictive value. In particular, extraversion and openness to experience reported a positive relation with SNS use, whereas emotional stability a negative one, implying that those showing negative emotionality, in general, tend to rely on SNSs more. Indeed, life satisfaction remained a strong negative predictor even after accounting for personality. As previously discussed, of particular relevance is openness to experience, as the role of this dimension is limited to the older cohort. As with emotional stability [12], other differences with the young media generation could be expected, further speculating on the novelty valence that SNSs might have for older adults as they need to learn to live with such tools, while being the normal mean of interaction for the youngsters.

### Gender Differences in Social Network Sites Use

Concerning gender differences, females were reported to have more friends on SNSs as compared with males. In addition, a study observed that for females, extraversion and openness to experience showed a positive relation and no association with emotional stability, with life satisfaction never of relevance [12]. As for males, the latter personality trait presented a negative relation with SNSs frequency of use, whereas extraversion a positive one, and openness to experience showed no significant relation. Further supporting the relation between gender and SNS use, 1 study reported, even thought of no particular significance, women to be more likely to post photos and update their status [4]. Life satisfaction did not play any predictive role, only after accounting for personality variables. These differences in SNS usage are because of the different motivations expressed by females and males. Still, considering belongingness need, women reported using SNSs to satisfy closeness and relational needs by showing a greater amount of connections with friends [15]. Particularly, females both with negative and positive collective self-esteem used SNSs to communicate, entertainment, and passing time [15]. Differently, males with negative or positive collective self-esteem used SNSs as an attempt at social compensation, using them to learn about others, for social identity gratification, and to seek social companionship [15].

## Discussion

### Principal Findings

SNSs are increasingly becoming of great relevance in people’s social life as in today’s society these kinds of platforms have crept into many daily activities, with almost everybody using it, still not in the same manner and for different reasons, for which of value has been the consideration of the FFM personality traits and motivation. This systematic review aimed to develop a cohesive outline of the past literature in relation to SNS use through the consideration of the factors influencing it, specifically motivation and personality traits as well as personal variables such as age and gender. Regarding personality traits, extraversion has been recognized as the main forerunner for SNS use and motivation for use by recreating on the Web real-life social dynamics; still, SNSs offer advantages also for the *socially awkward* [2]. Examples of such benefits are the asynchrony in communication, the lack of direct feedback, and the chance of acquiring information about others in an indirect and passive manner and the possibility of “ *manipulating* ” one’s own image [19]. Indeed, another relevant dimension is neuroticism, whose value has been speculated to be related to an attempt of compensating for their difficulty in real-life social contexts. In addition to individual differences, gender and age differences have been investigated. For the latter, openness to experiences has shown the greatest relevance as for adults and older adults SNSs are still perceived as a novelty. Differently, gender differences in SNS usage were observed to be the product of differences in motivation. Specifically, males use SNSs as social compensation tools, further supported by the negative relation with emotional stability, whereas females show as stronger motivator that of satisfying relational needs by seeking closeness [12]. Such findings can be considered also in relation to the dual-factors model of Facebook use in which authors identify 2 basic needs related to SNS use: the *need to belong* and the *need for self-presentation* [1,9]. Their value can be logically explained, as SNSs are indirect means of contact and communication, thus favoring both the sociable and the *socially awkward* individuals [1]. Throughout this review, what has been analyzed is SNS use and not abuse or addiction. Indeed, research on these 2 latter topics is inconsistent. As for *p* *sychopathology* and its relation with SNSs, as for addiction, further explorations are required, also focusing on the age differences possibly mediating the relation between SNS use and/or abuse and psychopathology. For example, the older population might present a greater risk for psychopathological repercussions of excessive SNS use, particularly for those already presenting a diathesis (eg, for anxiety disorders or depression), which could be even more drawn toward these new social tools. Moreover, future investigations should further consider the role of narcissism, whose relevance exceeds that of extraversion in regard to self-presentational motives [18]. It was hypothesized that SNSs are highly egocentric platforms reflecting an individualistic focus, and in this respect, it was underlined that such narcissistic tendencies and SNS use fuel each other in a way that requires further investigation, particularly as narcissism has been associated with poor impulse control and a lack of empathy [18]. Being this latter study conducted in Singapore, a quite westernized city-state, there appears to be the need for supplementary research on the differences between individualistic and collectivistic countries in SNS use, the motivation for use, and in the role and value of narcissism in the 2 societies. In this second decade of the 21st century, together with Facebook, the use of other platforms, such as *Twitter, Instagram, Snapchat,* and *Tumbler* and so on have become widespread. Still, they have not been considered by past literature, to the exclusion of Facebook, and as a consequence, not mentioned in this review. Indeed, this remains a limitation of many past studies and as such a suggestion for future research can be prompt for the consideration of the varied types of SNSs specifically, as offering a different possibility of use. For this reason, they attract different people, thus resulting in different motivations for use, to associations with different personality traits, and potentially with different psychopathologies.

### Conclusions

These results obtained through the systematic review of 9 articles, showed that, among the Big Five personality traits, extraversion appears to be the one better accounting for SNSs and motivation for use, favoring from an already satisfying real-life social network and competences, by transforming real-life relationships into Web ones. Indeed, SNSs are indirect means of contact and communication, encouraging both the sociable and the *socially awkward* individuals, such as to achieve information concerning others in a passive way and the possibility to manipulate of one’s own image. Thus, to counteract social passiveness, neuroticism was observed to be of great relevance. Differently, openness to experience was reported to be related to age differences, being of value only for the older cohort. The role of conscientiousness and agreeableness, on the other hand, was not sufficiently investigated. As for gender differences, results showed differences in the motivation for SNS use, with females reporting the need of seeking closeness, whereas males for social compensation purposes. In conclusion, SNS use seems to be associated to different motivations for use and to different personality traits, for which findings are still inconsistent. Therefore, future studies are still needed to further explore our conclusions.

## Appendix

Multimedia Appendix 1 Articles used in the literature review.

## Abbreviations

FFM: five-factor model
PRISMA: Preferred Reporting Items for Systematic Reviews and Meta-Analyses
SNS: social network site
