# Supplementary material for: Predisposing and Motivational Factors Related to Social Network Sites Use: Systematic Review
Source: JMIR Form Res. 2019 Jun 9;3(2):e12248. doi: 10.2196/12248 (PMC6592479; doi:10.2196/12248)
Supplement: Multimedia Appendix 1 [file formative_v3i2e12248_app1.doc]

Supplementary Table 1. The 9 articles for literature review.

| Authors, years, study | Sample (N), adult/adolescent, mean age (years) | Tools | Type of Social Networking Sites (SNS) | Main results |
| --- | --- | --- | --- | --- |
| Seidman  (2013) [14] | N=184, young adults, mean age: 19.51 | (1) Saucier's (1994) brief version of Goldberg’s Big Five markers, (2) Four scale for belongingness of Facebook were computed (2 for belongingness behavior and 2 for belongingness motivation), (3) Six scale for self-presentation of Facebook (2 for self-presentation behavior, 1 for attention seeking motivation, 3 for the different self-aspects expressed). | Facebook | (1) High agreeableness and neuroticism are the best predictors of belongingness motives and related behavior, (2) Extraversion is associated with a greater frequency of Facebook use to communicate, (3) Conscientious people are careful in their Web self-presentation, (4) Neuroticism is positively associated with the expression of ideal and hidden self-aspects, (5) Motivation behind the expression of the self-aspects mediates the relation between neuroticism and self-disclosure. |
| Allemand et al (2012) [16] | N=463, adolescents and young adults, mean age: 18.36 | (1) Facebook Intensity Scale (Ellison et al (2007), (2) The Rosenberg Self-Esteem Scale (RSE; Rosenberg, 1965), (3) The Experiences in Close Relationships-Revised (Fraley et al, 2000), (4) The five-factor model (Digman, 1990; McCrae and John, 1992), (5) Interpersonal Competence Scale (Buhrmester et al, 1988). | Facebook | (1) Extraversion is positively related to Facebook use, (2) Extraversion, agreeableness and openness to experience are positively related to competence in the initiation of interpersonal relationship. |
| Wang et al (2012) [13] | N=256, young adults, mean age: 20.15 | (1) Self-report on SNSs use, in particular on Renren website, (2) The Big Five Personality Inventory (John et al, 1991), (3) RSE (Rosenberg, 1965), (4) Narcissism Personality Inventory-16 (Ames et al, 2006), (5) The Sensation Seeking Scale (Zhao, 2004). | Renren website and SNSs in general | (1) Extraversion is positively related to number of friends, posting comments, self-photos, and updating status; (2) Neuroticism is positively related to updating one’s status as a means of self-expression, and neuroticism plays a role in information control; (3) Conscientiousness is positively related to SNSs use; (4) Agreeableness is positively related to writing comments on SNS and to the number of friends on SNSs; (5) Narcissism is positively related to posting photos and status updating. |
| Ong et al (2011) [18] | N=275, adolescents, mean age: 14.18 | (i) 12-item Narcissism Personality Questionnaire for Children-Revised (Ang and Raine, 2009), (2) NEO Five-Factor Inventory using the 12 item Extraversion subscale (Costa and McCrae, 1992), (3) Facebook use self-report, opened responses. | Facebook | (1) Narcissism predicts Facebook profile picture rating and the frequency of Facebook status updates (self-generated content), (2) Narcissism does not predict Facebook number of friends and Facebook photos over and above extraversion. |
| Nadkarni and Hofmann*,* Review (2011) [1] | young adults. | No tools used. | Facebook | (1) High level of extraversion, low self-esteem, high levels of neuroticism, narcissism, and low level of self-worth are associated with high Facebook use; (2) Frequent Facebook use is also associated with lower academic performance and probably with higher self-esteem and a sense of belonging; (3) Individuals with high level of narcissism (positive association between narcissism and Facebook use) and people with low levels of self-esteem tend to spend more than an hour a day on Facebook. |
| Kuss and Griffiths*,* Review (2011) [17] | adolescents and adults | No tools used. | Facebook, MySpace and SNS in general | (1) Females preferred to use SNSs to maintain contacts with actual friends rather than making new ones, as males did; (2) Females used SNSs for communication with peer group members, for entertainment, and passing time, whereas men tend to use it for social compensation, learning, and social identity gratification; (3) Social factors were more important motivators for SNSs usage than individual factors; collective self-esteem and group identification is positively related to peer group communication via SNSs; (4) Extraverts people seem to use SNSs for social enhancement, whereas introverts use it for social compensation, each of which appears to be related to higher usage, as does low conscientiousness and high narcissism. |
| Correa et al (2010) [12] | N=959, young adults and adults,  mean age: 46 | (1) The 10-items Personality Inventory (Gosling et al, 2003), (2) Satisfaction With Life Scale (Diener et al, 1985). | SNS in general | (1) Extraversion and openness to experiences are positively related to SNSs use, (2) Extravert men and women tend to be more frequent users of SNSs, (3) Extraversion was the strongest predictor of SNSs use, in particular in young adults, (4) Individuals with high level of neuroticism and negative affectivity are more likely to use SNSs. |
| Barker (2009) [15] | N=734, young adults , mean age: ≥18 | (1) Social Identity Gratifications Scale (Harwood, 1999), (2) Group Identification and Self-Esteem (Tropp and Wright, 1999). | Facebook, MySpace, Friendster | (1) Individuals with high collective self-esteem also present SNSs use to communicate with peer group members, (2) Positive collective self-esteem is also strongly correlated to entertainment and passing time, whereas negative collective self-esteem is related to the use of SNSs for social compensation, learning, and social identity gratification, (3) Males are more prone to look for social compensation and social identification as well as to learn about the *social* world through SNSs. |
| Ross et al (2009) [11] | N=97, young adults, mean age: 21.69 | (1) Facebook questionnaire (28-items; developed by the author), (2) Computer-mediated communication (CMC; Spitzberg, 2006), (3) NEO Personality Inventory-Revised (McCrae and Costa, 1992). | Facebook | (1) High extraversion individuals show greater Web group membership; (2) Extraversion is not significantly related to the number of Facebook friends, time spent on the Web, nor to the communicative features; (3) Neuroticism is not significantly related to the posting of personally identifying information nor to the communicative features; (4) Competency factors have an impact on Facebook use; (5) High CMC motivation is associated with greater time per day spent of Facebook. |
